# Supplementary material for: Sustained-input switches for transcription factors and microRNAs are central building blocks of eukaryotic gene circuits
Source: Genome Biol. 2013 Aug 23;14(8):R85. doi: 10.1186/gb-2013-14-8-r85 (PMC4054853; doi:10.1186/gb-2013-14-8-r85)
Supplement: Additional file 5 — HTML Browsable Motif Output. Zipped folder containing all WaRSwap and FANMOD motif output, viewable in a web browser. [file gb-2013-14-8-r85-S5.ZIP › HTML_browsable_motif_output/WaRSwap_all_tair9/sigs_WSWRSLDraw-2000_all.pvals.heatmaps.html/motif_id_46_011101002_tftype_all_upstream_-1000_0.html]

```
BG_MODEL = Rnew2
MOTIF_ID = 46_011101002
TF_TYPE = all
UPSTREAM = -1000_0


PVals
FN_0.2	FN_0.4	FN_0.6	FN_0.8
dg_60.genes	0.002	0.001	0.015	0.121
dg_70.genes	0.001	0.001	0.018	0.114
dg_80.genes	0.002	0.002	0.021	0.105

ZScores
FN_0.2	FN_0.4	FN_0.6	FN_0.8
dg_60.genes	3.049	3.191	2.41	1.155
dg_70.genes	3.017	3.113	2.279	1.23
dg_80.genes	2.994	3.09	2.281	1.261

StDevs
FN_0.2	FN_0.4	FN_0.6	FN_0.8
dg_60.genes	912483.579	182776.25	14417.481	650.779
dg_70.genes	929143.309	184714.978	14726.914	622.42
dg_80.genes	919413.804	189064.462	14882.394	621.937
```
